# External Treatment With Chinese Herbal Medicine for Chemotherapy-Induced Peripheral Neuropathy: A Systematic Review and Meta-Analysis

**DOI:** 10.3389/fphar.2022.764473

**Published:** 2022-02-18

**Authors:** Quan-yao Li, Fei-hong Cai, Ying Lu, Hui Liu, Xiao Wang, Fan-lian Li, Jun Shi

**Affiliations:** ^1^ Yueyang Hospital of Integrated Traditional Chinese and Western Medicine Affiliated to Shanghai University of Traditional Chinese Medicine, Shanghai, China; ^2^ School of Acupuncture-Moxibustion and Tuina, Shanghai University of Traditional Chinese Medicine, Shanghai, China; ^3^ Shanghai University of Traditional Chinese Medicine, Shanghai Traditional Chinese Medicine-Integrated Hospital, Shanghai, China

**Keywords:** traditional Chinese medicine, Chinese herbal medicine, chemotherapy-induced peripheral neuropathy, systematic review, meta-analysis

## Abstract

**Background:** Chemotherapy-induced peripheral neuropathy (CIPN) is a common adverse reaction of chemotherapy. Many studies have confirmed that traditional Chinese medicine (TCM) has unique advantages for treating CIPN. However, there is no standard TCM prescription in clinical practice or objective outcome index, and similar efficacy varies. Therefore, in this study, a systematic review of randomized controlled trials (RCTs) was performed to evaluate the clinical efficacy of external treatment with Chinese herbal medicine (CHM) for CIPN. This analysis provides evidence-based medical support for the use of CHM for external treatment of CIPN.

**Methods:** Relevant RCTs assessing CHM external treatment of CIPN were searched in nine electronic databases, including the China National Knowledge Infrastructure Database, China Biology Medicine Disc, China Science and Technology Journal Database, Wanfang Database, PubMed, Cochrane Library, MEDLINE, Web of Science, and OVID, from inception to July 2021. A meta-analysis was performed on these studies using RevMan5.3 software.

**Results:** Based on the inclusion and exclusion criteria, 33 clinical studies were included, while 1,354 studies were screened out. There were 2,356 patients in total, including 1,208 in the treatment group and 1,148 in the control group. In the treatment group, peripheral neurotoxicity rate, total effect rate, KPS score, TCM syndrome score and efficacy, pain NRS score, and pain relief rate were significantly improved compared with those of the control group (*p* < 0.01). Furthermore, the peroneal and median nerve conduction velocities were also improved compared with those in the control group (*p* < 0.05). By creating a funnel plot for the incidence of peripheral neurotoxicity and the total effect rate, we showed that the left and right sides were symmetrical, and that the publication bias was low.

**Conclusion:** CHM external treatment was found to be an effective method for treating CIPN as it significantly improved clinical symptoms and quality of life in patients with CIPN.

**Clinical Trial Registration:** identifier ChiCTR1900024617

## 1 Introduction

Chemotherapy-induced peripheral neuropathy (CIPN) refers to sensory or motor disorders of the extremities due to direct damage to the peripheral nervous system by chemotherapy drugs (Peng Xu et al., 2018). Clinical symptoms include symmetrical pain, numbness, and fine motor impairment at the extremities (Tian and Huang, 2017; Cao et al., 2019), but the main symptom is sensory impairment (Argyriou et al., 2012). CIPN can cause severe disability (Van Hecke et al., 2013), and its advanced development can limit physical activities. The incidence of CIPN closely correlates with the chemotherapy regimen, administration dosage, and evaluation methods (Zis et al., 2017; Avan et al., 2018), and it has been shown that in patients receiving drug combination, CIPN occurs in more than 38% of patients (Waseem et al., 2018). At present, CIPN is often relieved by reducing the dose of chemotherapeutics or by stopping the treatment, which seriously affects treatment efficacy and patient quality of life (Ma et al., 2018). However, there is no specific Western medicine treatment for CIPN. Neuropathic pain management and nutritional supplements are often applied but with poor curative effects (Staff et al., 2017). Therefore, it is vital to identify effective treatments for CIPN. As a traditional Chinese medicine (TCM) therapy, Chinese herbal medicine (CHM) has unique advantages in improving clinical symptoms, enhancing the curative effect of chemotherapy, and reducing its side effects (Liu et al., 2013). This study systematically evaluated the clinical efficacy of external treatment with CHM for CIPN aiming to provide higher-quality clinical evidence and guidance on CHM external treatment of CIPN.

## 2 Materials and Methods

### 2.1 Search Strategy

Database retrieval strategies were formulated in accordance with the requirements of the Cochrane Systematic Review Manual, and computer retrieval was the main method. A comprehensive literature search was conducted in the China National Knowledge Infrastructure (CNKI), China Biology Medicine Disc (CBM), China Science and Technology Journal (VIP), Wanfang (WANFANG), PubMed, Cochrane Library, MEDLINE, Web of Science, and OVID databases. The following search terms were used as the search strategy and were modified according to different databases: “chemotherapy-induced peripheral neuropathy,” “chemotherapy-induced peripheral neurotoxicity,” “CIPN,” “peripheral neuropathy,” “peripheral neurotoxicity,” “drugs,” “Chinese herbal,” and traditional Chinese medicine.” In addition, we manually retrieved relevant articles and clinical studies to obtain as much literature as possible. No language or status restriction was set in this review.

### 2.2 Inclusion Criteria

The following components were included in the present study:

1) Types of studies: Randomized controlled clinical trials (RCTs) that evaluated the efficacy of external treatment with CHM for chemotherapy-induced peripheral neurotoxicity were included.
2) Types of participants: Patients who were pathologically or cytologically diagnosed with malignancy and developed peripheral neuropathy after chemotherapy were considered. No restriction on gender, age, or nationality was set.
3) Types of interventions: Patients in the control group were treated with conventional chemotherapy regimens or further given conventional Western medicine and placebo treatments.
4) Patients in the treatment group were treated with external TCM treatment (external TCM washing, wet dressing, and/or foot bath) in addition to conventional chemotherapy or control treatment.

The following outcome measures were considered:

1) Primary outcomes: incidence of CIPN, overall response rate, nerve conduction velocity, Karnofsky performance score (KPS), TCM syndrome score (Dai et al., 2017), and TCM syndrome effect (Zheng, 2002); clinical recovery: clinical symptoms and signs disappeared or almost disappeared, and TCM syndrome score decreased ≥90%; significant efficacy: clinical symptoms and signs disappeared, improved significantly, and TCM syndrome score were reduced by 70%; efficacy: clinical symptoms and signs are improved, and TCM syndrome score was reduced by 30%; inefficacy: the disappearance of clinical symptoms and signs was not significant, and the TCM syndrome score was reduced by less than 30%. Calculation formula: (score before treatment—score after treatment) ÷ score before treatment × 100%
2) Secondary outcomes: numeric rating scales, myelosuppression, and analgesic rate.

### 2.3 Exclusion Criteria

The following components were excluded in the present study:

1) Non-randomized controlled trials, case studies without a control group, for example, review, meta-analysis, discussion, conference, cell tissue or animal experiments, and other non-clinical trials.
2) Self–cross-control study.
3) Literature that did not meet outcome measures or failed to present valid data.
4) Repeated publication or repeated detection of literature.
5) Participants whose neurological disorders resulted from electrolyte disorders and other systemic diseases, such as severe cervical spondylosis, severe intervertebral disc compression, severe diabetes, and other non-chemotherapeutic factors.
6) Participants with hand and foot skin diseases and a history of drug allergy.
7) Intervention not including external TCM treatment.

### 2.4 Study Selection and Data Extraction

Studies were preliminarily independently screened by two researchers based on the inclusion and exclusion criteria, primarily through the title, abstract, and keywords. Then, the full text was read for re-screening, and the results were cross-checked. If a difference occurred, a third party made the final decision. If a decision was not reached, relevant professionals were consulted. The following information was extracted: author, year of publication, number of participants, grouping methods, interventions, and outcome measures.

### 2.5 Literature Quality Evaluation

A new “risk of bias assessment” tool developed by methodologists, editors, and systematic reviewers was used to evaluate the quality of the included literature as recommended by the Cochrane Collaboration (Higgins et al., 2011). This includes six aspects: 1) random allocation scheme; 2) allocation concealment; 3) blinding of participants and personnel; 4) integrity of the data; 5) selective reporting of research results; and 6) other sources of bias. Each field was assessed as “yes” (low ROB; risk of bias), “no” (high ROB), or “unclear” (unclear ROB).

### 2.6 Data Analysis

Review Manager (RevMan, Version 5.3, Copenhagen: The Nordic Cochrane Center, The Cochrane Collaboration, 2014) was utilized to conduct the data analysis. A heterogeneity test was performed on the results of each included study. No statistical significance (*p* > 0.05, I^2^ < 50%) indicated that there was no significant statistical heterogeneity in the included studies, and a fixed-effects model was used for the analysis. Statistical significance (*p* < 0.05, I^2^ > 50%) indicated that heterogeneity existed in the included studies, and further analysis was needed to determine the source of heterogeneity and whether a random-effects model could be used for the analysis. The odds ratios (ORs) and 95% confidence intervals (CIs) were utilized for efficacy analysis of dichotomous variables, while standardized mean difference (SMD) and 95% CIs were utilized for efficacy analysis of continuous variables.

## 3 Results

### 3.1 Literature Retrieval Results

**FIGURE 1:**
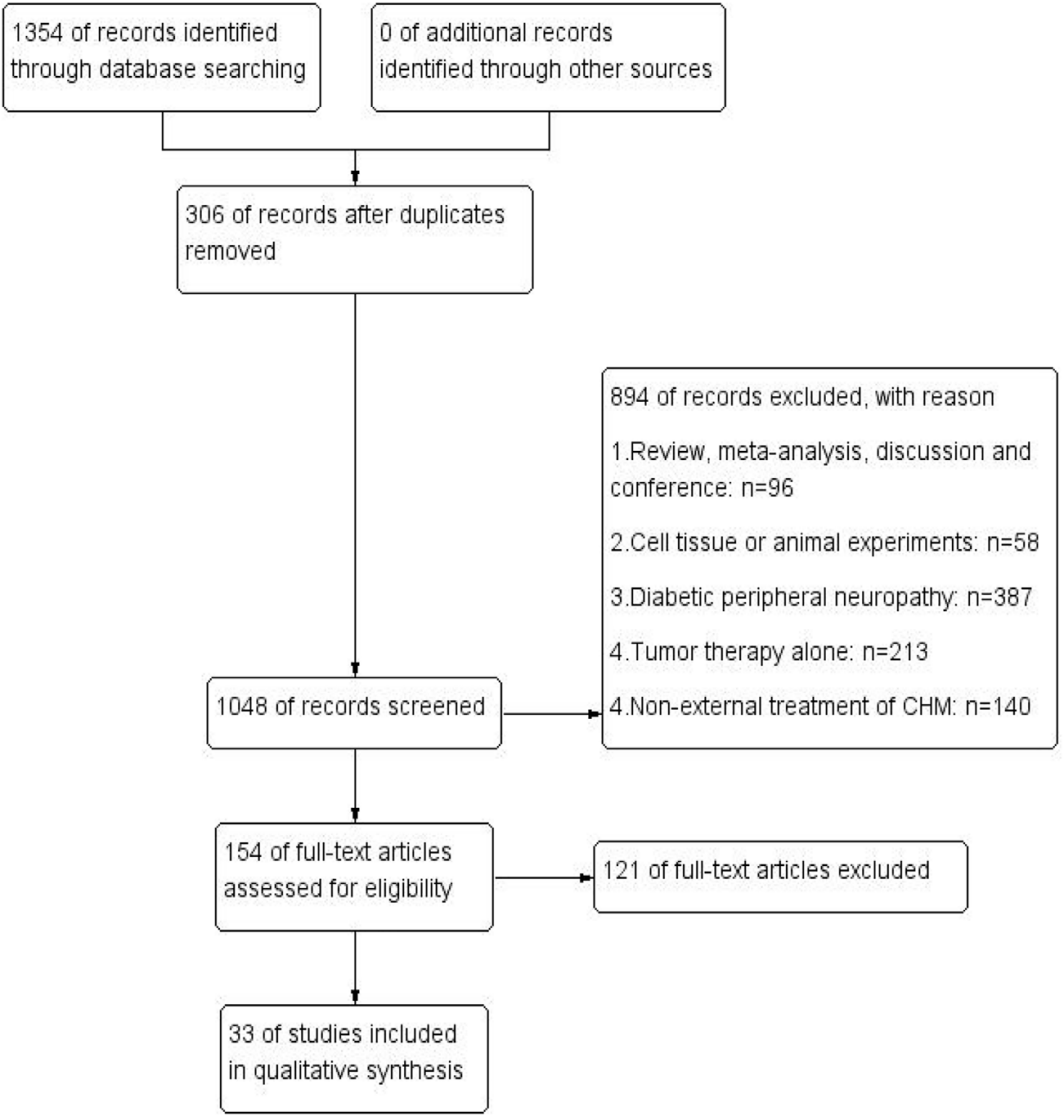
Flowchart of literature identification and screening. PubMed (*n* = 49), Cochrane library (*n* = 44), Web of Science (*n* = 79), MEDLINE (*n* = 3), OVID (*n* = 8), CNKI (*n* = 234), CBM (*n* = 528), VIP (*n* = 40), and WANFANG (*n* = 369).

The process of literature identification and screening is described in the flowchart (Figure 1). In total, 1,354 related studies were derived from electronic databases. After removing 306 duplicates, 1,048 studies were rescreened through titles and abstracts. Then, 894 were excluded, while 154 remained to be assessed in full text. Ultimately, 33 eligible RCTs that satisfied the inclusion and exclusion criteria were included.

### 3.2 Basic Features and Quality Evaluation

In total, 33 RCTs with a total of 2,356 patients were included. There were 1,208 patients in the treatment group and 1,148 in the control group. The baseline criteria (age, sex, and other factors) in each included study were balanced, indicating comparability between the treatment and control groups (Table 1).

**TABLE 1:** Basic information about the included literature.

Included literature	Sample size		Baseline (age, sex, and other factors)	Intervention		Outcome measures
	Control group	Treatment group		Control group	Treatment group	
Qiao, (2019)	30	30	Coincide	Mecobalamin	Oral CHM + CHM external washing	1, 2, 6, 7, 9
Li et al. (2020)	39	40	Coincide	Chemotherapy + warm water soak	Chemotherapy + CHM wet washing	1, 3, 7, 8
Fan, (2019)	25	25	Coincide	Mecobalamin	Fumigation of CHM	1, 2
Zhu, (2017)	28	22	Coincide	Chemotherapy	Chemotherapy + CHM external washing	1, 4
Yuan, (2015)	28	27	Coincide	Chemotherapy + warm water soak	Chemotherapy + CHM washing out	1, 6
Yan, (2019)	36	36	Coincide	Mecobalamin	Mecobalamin + CHM external washing	1, 2, 4, 5
Yu and Xu, (2019)	26	27	Coincide	Chemotherapy + glutathione + warm water soak	Chemotherapy + Oral CHM + CHM external washing	1, 4, 8
Ke et al. (2016)	20	20	Coincide	Mecobalamin	Mecobalamin + CHM external washing	2, 3
Song, (2017)	33	36	Coincide	Chemotherapy + mecobalamin	Chemotherapy + mecobalamin +Oral CHM + CHM external washing	1, 6
Zhuang, (2019)	30	30	Coincide	Chemotherapy + warm water soak	Chemotherapy + CHM external washing	1, 6, 8
Gu et al. (2018)	45	45	Coincide	Mecobalamin	Mecobalamin + CHM external washing	3, 9
Cai, (2016)	25	25	Coincide	Chemotherapy	Chemotherapy + CHM external washing	1, 8
Li et al. (2020)	31	34	Coincide	Chemotherapy	Chemotherapy + CHM fumigation and external washing	1, 4, 8
Baoyi Wang, (2020)	24	27	Coincide	Mecobalamin + placebo	Mecobalamin + external application of CHM	1, 7
Wu, (2011)	30	30	Coincide	Chemotherapy	Chemotherapy + CHM external washing	1, 8
Gu, (2020)	29	28	Coincide	Mecobalamin	Mecobalamin + CHM external washing	1, 2, 4, 5, 6
Wei, (2014)	31	33	Coincide	Cobamamide	CHM External washing	2, 6
Jing-yu Xu et al. (2018)	31	36	Coincide	Mecobalamin + placebo	Mecobalamin + gel for external use	2, 3, 5
Li Wang, (2020)	39	38	Coincide	Chemotherapy + mecobalamin	Chemotherapy + CHM external washing	1, 2, 4, 5, 6
He and Li, (2019)	48	49	Coincide	Mecobalamin	Mecobalamin + CHM external washing	1, 4, 5
Deng, (2014)	51	54	Coincide	Chemotherapy	Chemotherapy + CHM external washing	1, 8
Tan and Qi, (2015)	30	33	Coincide	Mecobalamin	Mecobalamin + Oral CHM + CHM external washing	1, 2, 4, 7, 9
Xiong et al. (2017)	48	52	Coincide	Auricular point	Auricular point + CHM external washing	1, 2, 4
Zhu, (2017)	42	42	Coincide	Vitamin B1 + mecobalamin	Oral CHM + CHM external washing	1, 2
Yao et al. (2019)	93	102	Coincide	Warm water soak	Chemotherapy + CHM soak	3, 5
Yang et al. (2018)	30	30	Coincide	Chemotherapy+ cobamamide	Chemotherapy + cobamamide + TCM foot bath	2, 3
Gao et al. (2015)	32	30	Coincide	Warm water bath	CHM external washing	1, 2
Ma and Shan, (2017)	30	30	Coincide	Chemotherapy+ mecobalamin	Chemotherapy + TCM foot bath	1, 4
Yao, (2020)	30	30	Coincide	Mecobalamin	Mecobalamin + CHM external washing	1, 4, 6
Guo, (2015)	33	32	Coincide	Warm water soak	CHM fumigation	2, 4, 7
Zhang, (2019)	22	23	Coincide	Placebo	CHM fumigation	1, 2, 8
Lou et al. (2014)	34	68	Coincide	Placebo	CHM external washing	1, 2, 7, 9
Pan et al. (2021)	45	44	Coincide	Mecobalamin	Oral CHM + CHM external washing	2, 5

Note: (1) incidence rate of CIPN; (2) overall response rate; (3) nerve conduction velocity; (4) Karnofsky performance score (KPS); (5) TCM syndrome score; (6) TCM syndrome effect; (7) numeric rating scales; (8) myelosuppression; (9) analgesic rate.

**FIGURE 2:**
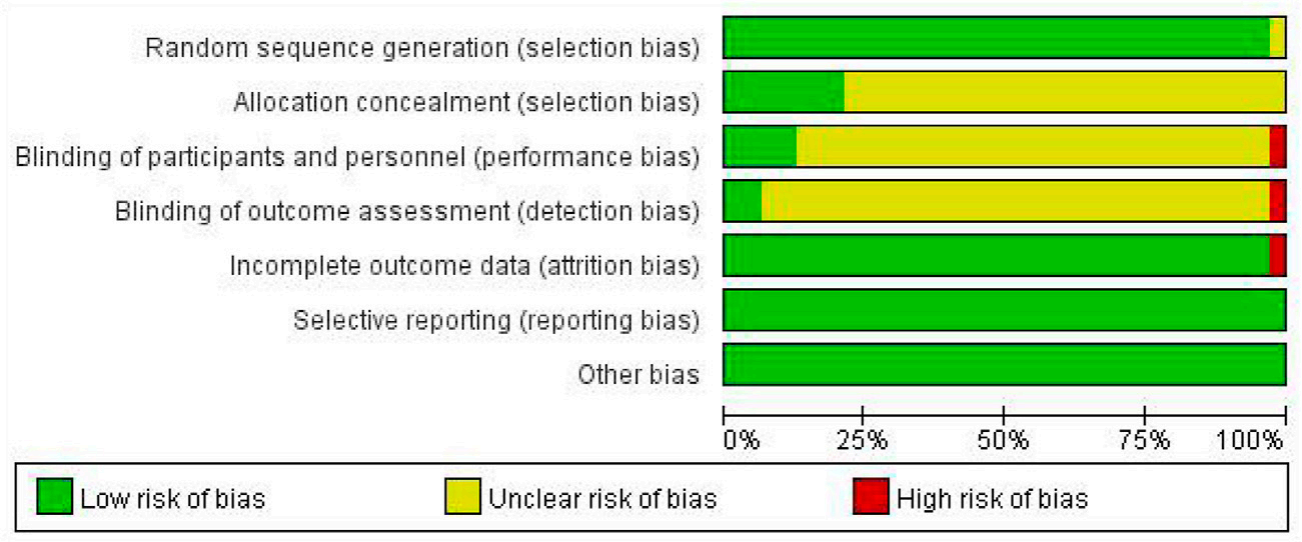
Risk-of-bias graph.

**FIGURE 3:**
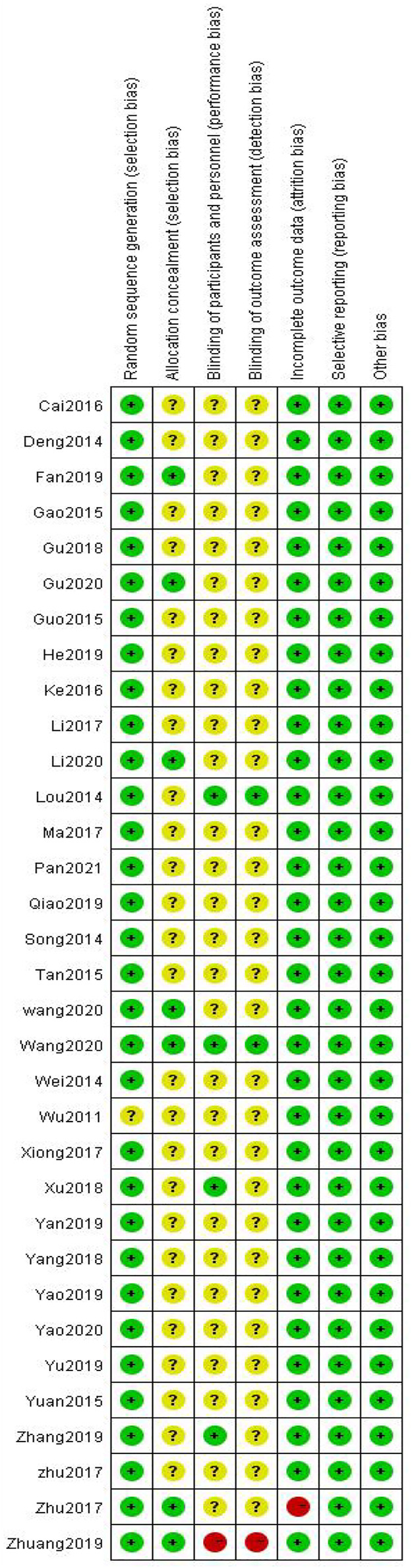
Risk-of-bias summary.

Among the 33 included studies, 20 applied a random-number table for grouping (Deng, 2014; Wei, 2014; Guo, 2015; Tan and Qi, 2015; Yuan, 2015; Ke et al., 2016; Zhu et al., 2017; Jing-yu Xu et al., 2018; Gu et al., 2018; Yang et al., 2018; He and Li, 2019; Yao et al., 2019; Yu and Xu, 2019; Zhang, 2019; Zhuang, 2019; Li Wang, 2020; Gu, 2020; Li et al., 2020; Yao, 2020; Pan et al., 2021); 10 only mentioned random allocation (Gao et al., 2015; Cai, 2016; Li and Cai, 2017; Ma and Shan, 2017; Song, 2017; Zhu, 2017; Fan, 2019; Qiao, 2019; Yan, 2019; Baoyi Wang, 2020); two used a number-parity method and stratified random-block method (Lou et al., 2014; Xiong et al., 2017); and one study did not provide any information on this aspect (Wu, 2011). Six studies used allocation concealment (Fan, 2019; Zhuang, 2019; Li Wang, 2020; Baoyi Wang, 2020; Gu, 2020; Li et al., 2020), and the remaining studies did not mention the use of allocation concealment. Five studies involved blinding (Lou et al., 2014; Zhu, 2017; Jing-yu Xu et al., 2018; Zhang, 2019; Baoyi Wang, 2020); one did not (Zhuang, 2019); and the remaining did not mention blinding (Zhu, 2017). All of the 33 studies fully reported the results without any selection or other bias, while only one study was incomplete due to subject dropout (Figures 2,3).

### 3.3 Results of the Meta-analysis

#### 3.3.1 Primary Outcomes

##### 3.3.1.1 Incidence of Peripheral Neurotoxicity

**FIGURE 4:**
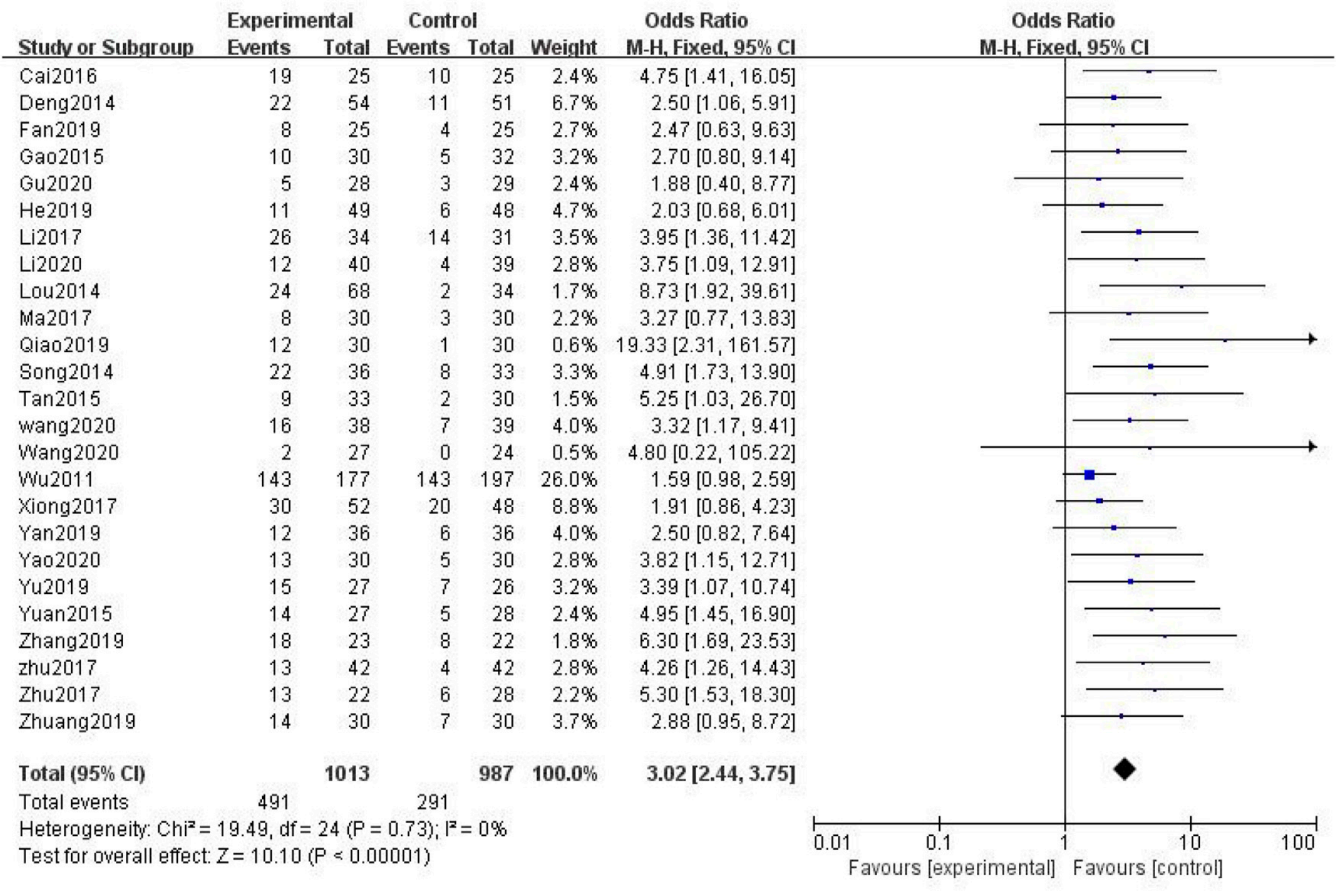
Incidence of peripheral neurotoxicity.

In total, 25 studies with 2,000 patients were included (Wu, 2011; Deng, 2014; Lou et al., 2014; Gao et al., 2015; Tan and Qi, 2015; Yuan, 2015; Cai, 2016; Li and Cai, 2017; Ma and Shan, 2017; Song, 2017; Xiong et al., 2017; Zhu, 2017; Zhu et al., 2017; Fan, 2019; He and Li, 2019; Qiao, 2019; Yan, 2019; Yu and Xu, 2019; Zhang, 2019; Zhuang, 2019; Li Wang, 2020; Baoyi Wang, 2020; Gu, 2020; Li et al., 2020; Yao, 2020), with 1,013 in the treatment group and 987 in the control group. The heterogenicity test showed homogeneity among the 25 studies (*p* > 0.05, I^2^ = 0%). Therefore, the fixed-effect model was used for data analysis. The meta-analysis showed a statistically significant difference in the incidence of peripheral neurotoxicity between the treatment and control groups (OR = 3.02, 95% CI [2.44, 3.75], *p* < 0.01, Figure 4).

##### 3.3.1.2 Overall Response Rate

**FIGURE 5:**
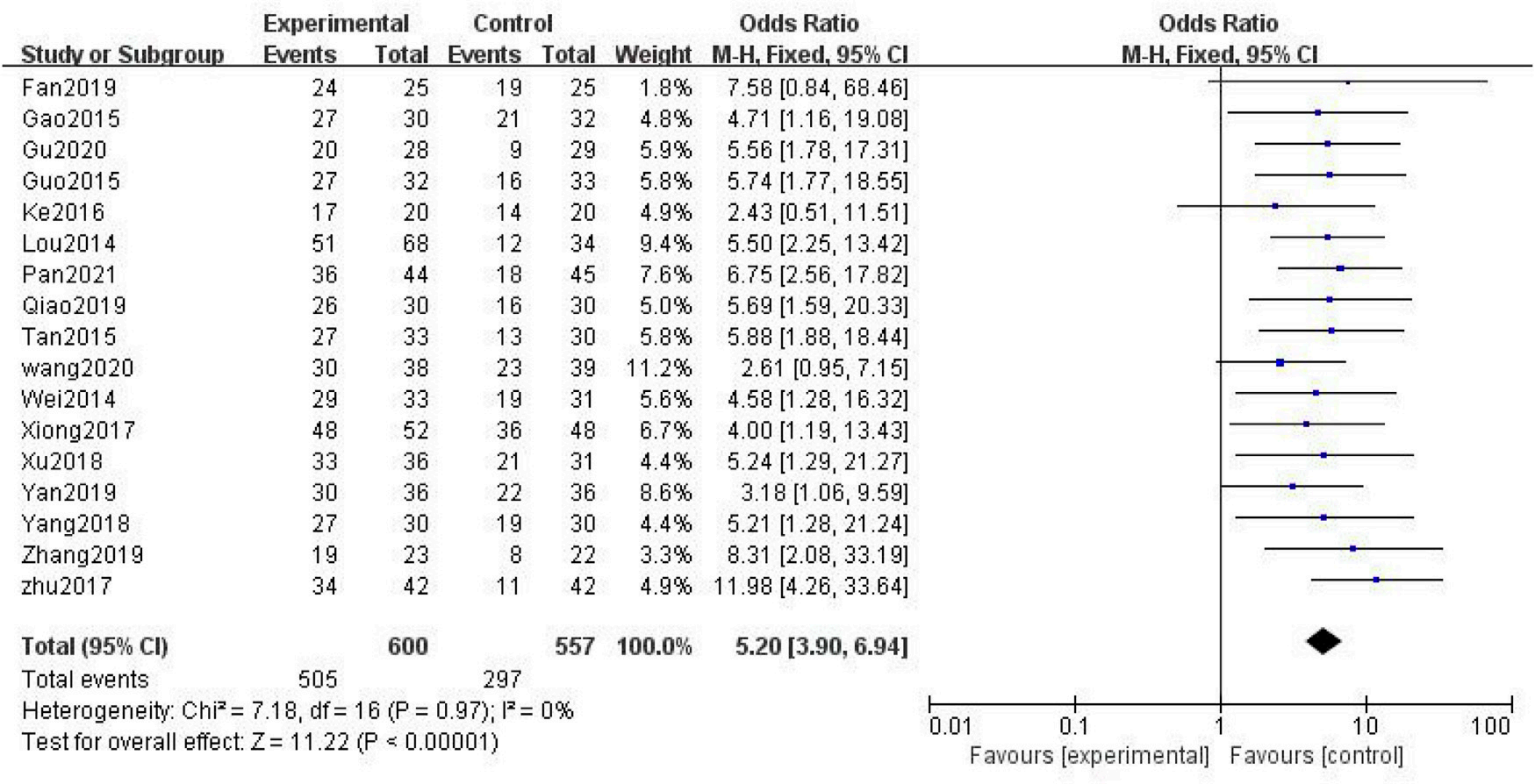
Overall response rate.

In total, 17 studies with 1,157 patients were included (Lou et al., 2014; Wei, 2014; Gao et al., 2015; Guo, 2015; Tan and Qi, 2015; Ke et al., 2016; Xiong et al., 2017; Zhu et al., 2017; Jing-yu Xu et al., 2018; Yang et al., 2018; Fan, 2019; Qiao, 2019; Yan, 2019; Zhang, 2019; Li Wang, 2020; Gu, 2020; Pan et al., 2021), with 600 in the treatment group and 557 in the control group. The heterogenicity test showed homogeneity among the 17 studies (*p* > 0.05, I^2^ = 0%). Therefore, the fixed-effect model was used for data analysis. The meta-analysis showed a statistically significant difference in the overall response rate between the treatment and control groups (OR = 5.20, 95% CI [3.90, 6.94], *p* < 0.01, Figure 5).

##### 3.3.1.3 Peroneal Nerve Conduction Velocity

**FIGURE 6:**
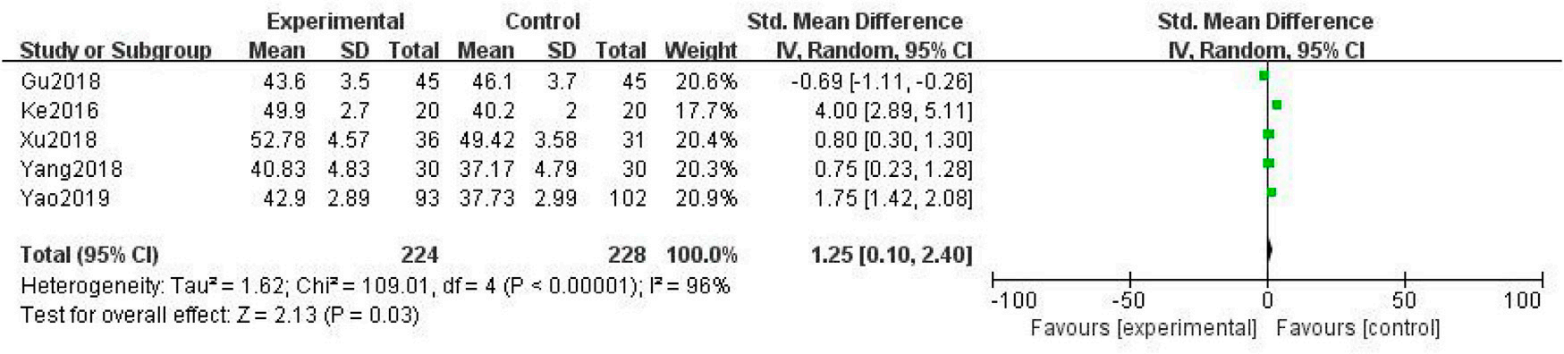
Peroneal nerve conduction velocity.

In total, five studies with 452 patients were included (Ke et al., 2016; Jing-yu Xu et al., 2018; Gu et al., 2018; Yang et al., 2018; Yao et al., 2019), with 224 in the treatment group and 228 in the control group. The heterogenicity test showed homogeneity among the five studies (*p* < 0.05, I^2^ = 96%). Therefore, the random-effect model was used for data analysis. The meta-analysis showed a statistically significant difference in the peroneal nerve conduction velocity between the treatment and control groups (SMD = 1.25, 95% CI [0.10, 2.40], *p* < 0.05, Figure 6).

##### 3.3.1.4 Median Nerve Conduction Velocity

**FIGURE 7:**
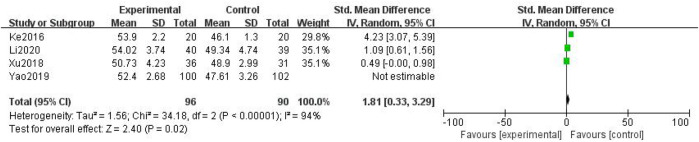
Median nerve conduction velocity.

In total, four studies with 388 patients were included (Jing-yu Xu et al., 2018; Yao et al., 2019; Gu, 2020; Li et al., 2020), with 196 in the treatment group and 192 in the control group. The heterogenicity test showed homogeneity among the four studies (*p* < 0.05, I^2^ = 94%). Therefore, the random-effect model was used for data analysis. The meta-analysis showed a statistically significant difference in the median nerve conduction velocity between the treatment and control groups (SMD = 1.81, 95% CI [0.33, 3.29], *p* < 0.05, Figure 7).

##### 3.3.1.5 KPS

**FIGURE 8:**
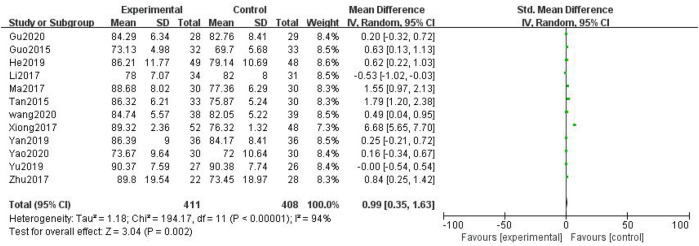
Karnofsky performance score.

In total, 12 studies with 819 patients were included (Guo, 2015; Tan and Qi, 2015; Li and Cai, 2017; Ma and Shan, 2017; Xiong et al., 2017; Zhu, 2017; He and Li, 2019; Yan, 2019; Yu and Xu, 2019; Li Wang, 2020; Gu, 2020; Yao, 2020), with 411 in the treatment group and 408 in the control group. The heterogenicity test showed homogeneity among the 12 studies (*p* < 0.05, I^2^ = 94%). Therefore, the random-effect model was used for data analysis. The meta-analysis showed a statistically significant difference in the KPS between the treatment and control groups (SMD = 0.99, 95% CI [0.35, 1.63], *p* < 0.01, Figure 8).

##### 3.3.1.6 Traditional Chinese Medicine Syndrome Score

**FIGURE 9:**
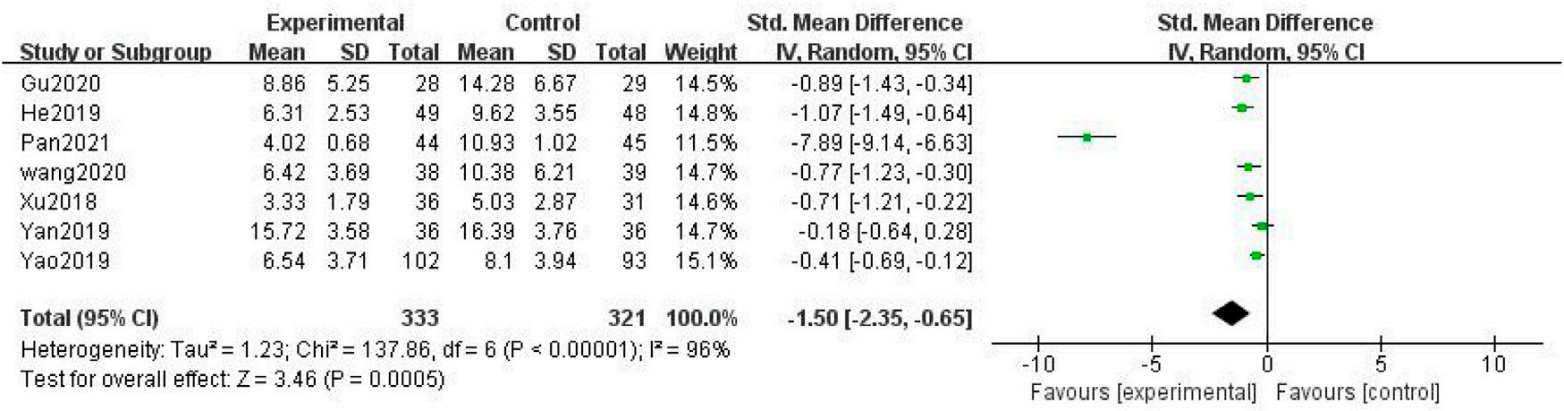
TCM syndrome score.

In total, seven studies with 654 patients were included (Jing-yu Xu et al., 2018; He and Li, 2019; Yan, 2019; Yao et al., 2019; Li Wang, 2020; Gu, 2020; Pan et al., 2021), with 333 in the treatment group and 321 in the control group. The heterogenicity test showed homogeneity among the seven studies (*p* < 0.05, I^2^ = 96%). Therefore, the random-effect model was used for data analysis. The meta-analysis showed a statistically significant difference in the TCM syndrome score between the treatment and control groups (SMD = –1.50, 95% CI [–2.35, –0.65], *p* < 0.01, Figure 9).

##### 3.3.1.7 Traditional Chinese Medicine Syndrome Effect

**FIGURE 10:**
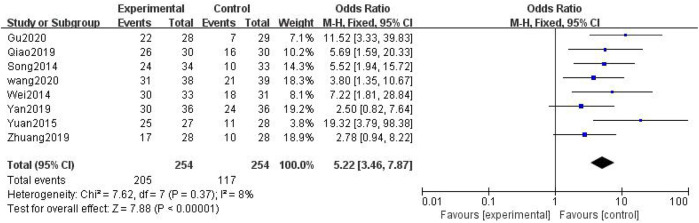
TCM syndrome effect.

In total, eight studies with 508 patients were included (Deng, 2014; Wei, 2014; Guo, 2015; Tan and Qi, 2015; Yuan, 2015; Song, 2017; Zhu et al., 2017; Yang et al., 2018; He and Li, 2019; Qiao, 2019; Yan, 2019; Yao et al., 2019; Zhang, 2019; Zhuang, 2019; Li Wang, 2020; Gu, 2020; Yao, 2020; Pan et al., 2021), with 254 in the treatment group and 254 in the control group. The heterogenicity test showed homogeneity among the eight studies (*p* > 0.05, I^2^ = 8%). Therefore, the fixed-effect model was used for data analysis. The meta-analysis showed a statistically significant difference in the TCM syndrome effect velocity between the treatment and control groups (OR = 5.22, 95% CI [3.46, 7.87], *p* < 0.01, Figure 10).

#### 3.3.2 Secondary Outcomes

##### 3.3.2.1 Numerical Rating Scale

**FIGURE 11:**
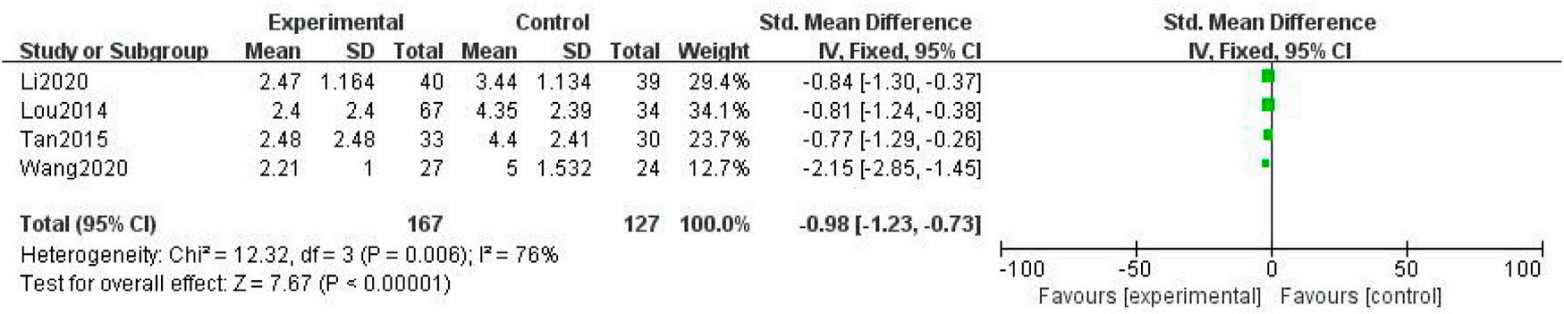
Numerical rating scale.

In total, four studies with 294 patients were included (Lou et al., 2014; Tan and Qi, 2015; Baoyi Wang, 2020; Li et al., 2020), with 167 in the treatment group and 127 in the control group. The heterogenicity test showed homogeneity among the four studies (*p* > 0.05, I^2^ = 76%). Therefore, the fixed-effect model was used for data analysis. The meta-analysis showed a statistically significant difference in the numerical rating scale between the treatment and control groups (SMD = –0.98, 95% CI [–1.23, –0.73], *p* < 0.01, Figure 11).

##### 3.3.2.2 Myelosuppression

**FIGURE 12:**
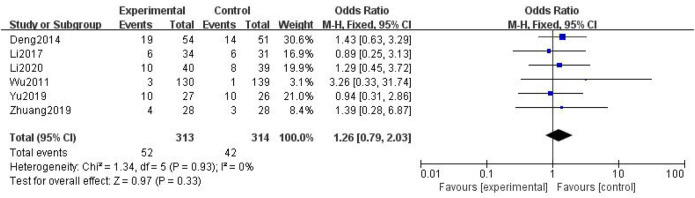
Myelosuppression.

In total, six studies with 627 patients were included (Wu, 2011; Deng, 2014; Li and Cai, 2017; Yu and Xu, 2019; Zhuang, 2019; Li et al., 2020), with 313 in the treatment group and 314 in the control group. The heterogenicity test showed homogeneity among the four studies (*p* > 0.05, I^2^ = 0%). Therefore, the fixed-effect model was used for data analysis. The meta-analysis showed no statistically significant difference in myelosuppression between the treatment and control groups (OR = 1.26, 95% CI [0.79, 2.03], *p* > 0.05, Figure 12).

##### 3.3.2.3 Analgesic Rate

**FIGURE 13:**
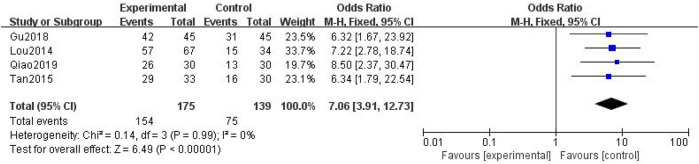
Analgesic rate.

In total, four studies with 314 patients were included (Lou et al., 2014; Tan and Qi, 2015; Gu et al., 2018; Qiao, 2019), with 175 in the treatment group and 139 in the control group. The heterogenicity test showed that there was homogeneity among the four studies (*p* > 0.05, I^2^ = 0%). Therefore, the fixed-effect model was used for data analysis. The meta-analysis showed a statistically significant difference in the analgesic rate between the treatment and control groups (OR = 7.06, 95% CI [3.91, 12.73], *p* < 0.01, Figure 13).

#### 3.3.3 Sensitivity Analysis

When heterogeneity was identified among studies in the outcome index, the causes of heterogeneity were analyzed. Examination of the impact of each study on this meta-analysis and sensitivity analysis was conducted; each study was eliminated to calculate the heterogeneity and effective size of the remaining studies.

#### 3.3.4 Publication Bias Assessment

**FIGURE 14:**
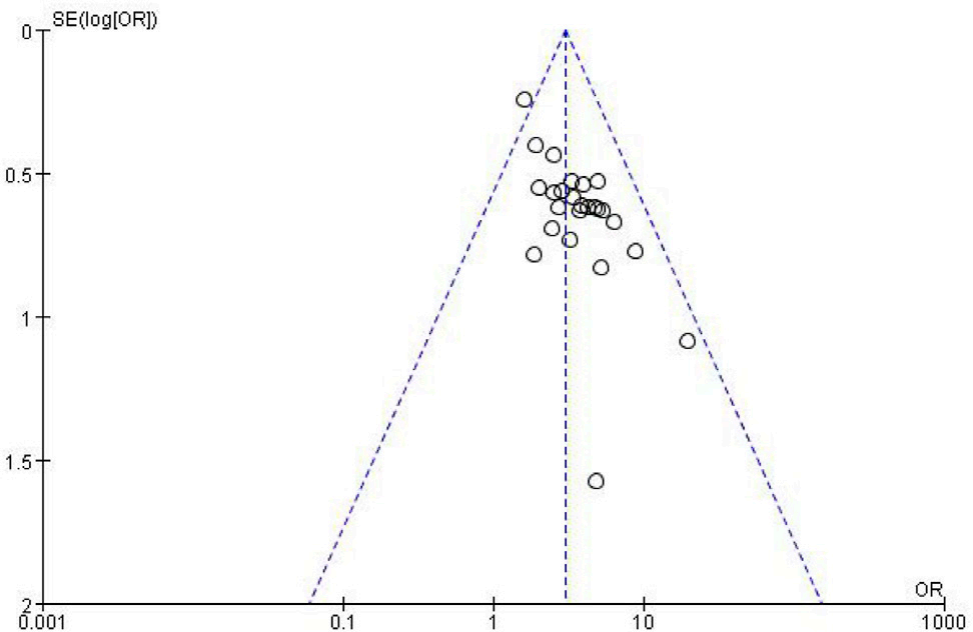
Peripheral neurotoxicity funnel plot.

**FIGURE 15:**
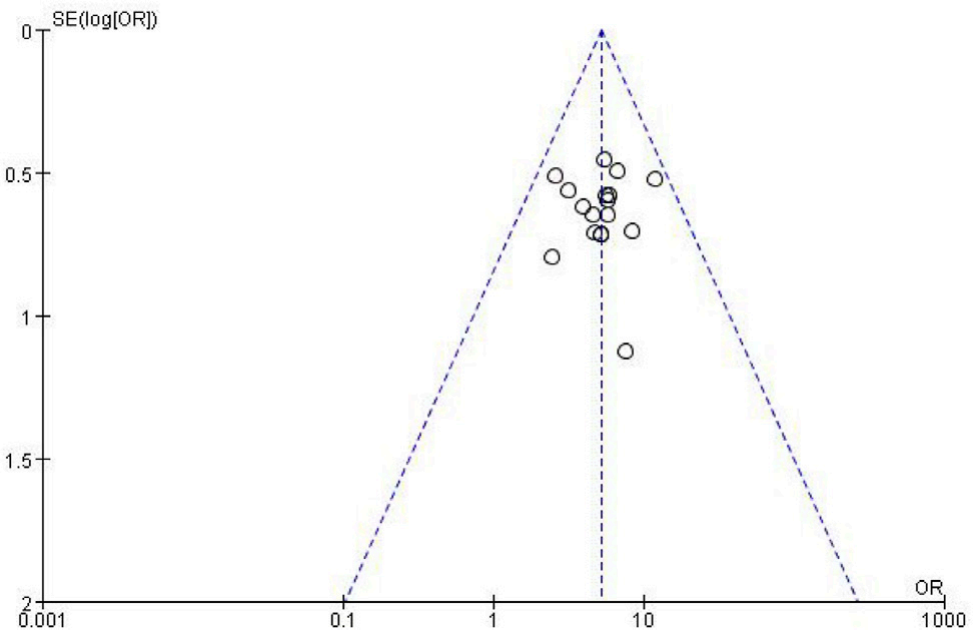
Overall response rate funnel plot.

The funnel plots for the incidence of peripheral neurotoxicity and the overall effect rate (Figures 14, 15) showed that the left and right sides were symmetric, indicating low publication bias. The results suggest that CHM external treatment decreased the incidence of peripheral neurotoxicity, increased clinical efficacy, and improved quality of life in patients with CIPN.

## 4 Discussion

CIPN is characterized by limb pain and numbness, which is referred to as “Bi syndrome” in TCM. Chemotherapeutic drugs belong to “toxic evil” in TCM; this is equivalent to anticancer CHM (Ren, 2015) and can easily damage the healthy qi of the human body. In addition, cancer patients lack essence and healthy qi, resulting in a deficiency of qi and blood, phlegm congealing, and blood stasis (Ma and Huo, 2019). Therefore, the basic principles of treatment should focus on tonifying qi, replenishing blood, promoting blood circulation, and dredging collaterals (Ji and Wu, 2016).

CHM external and internal treatments show similar efficacy with different features. CHM external treatment is based on the meridian theory, which can strengthen the penetration of drugs through the skin to the interior of the body. It not only helps the effective CHM ingredients to directly cure the illness and better relieve symptoms but also promotes blood circulation, improves immunity, and regulates the function of the viscera (Wang and Zhu, 2015). Furthermore, it solves the problem of a poor curative effect when patients fail to take oral medications for various reasons (Yan, 2019). The treatment reflects holism in TCM (Liu and Zuo, 2012). Previous studies have indicated that external treatment with CHM can effectively decrease the incidence of peripheral neurotoxicity, improve clinical efficacy and clinical symptoms, prevent the occurrence of adverse reactions, and improve patient quality of life (Ji et al., 2017). Li et al. (2020) found that wet application of CHM can prevent the occurrence of peripheral neurotoxicity, improve nerve conduction velocity, reduce NRS score, and effectively relieve pain symptoms. Ye and Chen (2019) have found that TCM fumigation and washing can significantly reduce the incidence of peripheral neurotoxicity, effectively improve adverse reactions such as bone marrow suppression, and improve quality of life, further confirming the effectiveness and safety of CHM for the treatment of CIPN.

Among the 33 included studies, Guizhi was the most frequently used CHM followed by Huangqi and Honghua, which is consistent with CIPN’s previous treatment principle of “benefiting qi, promoting blood circulation, removing blood stasis, warming Yang, and dredging collaterals” (Zheng et al., 2015). Guizhi lubricates the joints and warms the meridian, curing numbness and pain in the limbs. Previous studies have shown that Guizhi exhibits anticancer activity and promotes vascular protection by accelerating blood flow and inhibiting platelet aggregation and thrombosis (Huang et al., 2007). Huangqi can dilate blood vessels and improve peripheral blood supply, thereby promoting limb blood circulation (Chen, 2018). Honghua can activate blood, unblock meridians, disperse blood stasis, and relieve pain. Modern pharmacology shows that Honghua can protect vascular endothelial cells, inhibit vascular smooth muscle, nourish nerves, and improve nerve conduction velocity (Shi and Li, 2017).

In total, 33 studies were included in this study. The results showed that the treatment group had lower incidence of peripheral neurotoxicity and greater pain relief (*p* < 0.01), overall response rate, KPS, TCM syndrome effect (*p* < 0.01), peroneal nerve conduction velocity, and median nerve conduction velocity (*p* < 0.05), and reduced TCM syndrome score and NRS (*p* < 0.01) than that of the control group. However, there was no difference in myelosuppression between the two groups.

In summary, the treatment of CIPN using TCM showed preventive and control effects by decreasing the incidence of peripheral neurotoxicity, alleviating clinical symptoms, and improving the quality of life. However, there was no significant improvement in bone marrow inhibition. Bone marrow inhibition is a common adverse reaction in chemotherapy patients; it is caused by various mechanisms and leads to decline in the activity and function of hematopoietic stem cells in the bone marrow, resulting in inability to produce enough blood cells. It manifests as a decrease in white blood cells and platelet counts, resulting in a decline in the body’s immunity, and thus significantly affecting the compliance of chemotherapy patients and chemotherapy efficacy. Therefore, the results of this study suggest that TCM should be selected purposely based on the characteristic of bone marrow inhibition, which has important clinical significance. In addition, although the safety of Chinese medicine is high, and adverse reactions are relatively rare, attention is still needed. In the process of Chinese medicine treatment, doctors should pay close attention to the patient’s vital signs and physical condition. If discomfort exists, treatment should be immediately stopped, and appropriate measures should be taken. When dizziness or panic occurs, windows should be opened for ventilation, and patients should rest in bed. When allergic reactions occur, patients should take anti-allergy drugs under the guidance of a doctor and avoid scratching their skin.

## 5 Limitations

This study systematically evaluated the clinical efficacy of external CHM treatment of CIPN. CHM external treatment significantly reduced the incidence of peripheral neurotoxicity and improved clinical symptoms and quality of life. However, there were also some limitations to this study. First, cancer patients often suffer from a deficiency of qi and blood. Qi stagnation and blood stasis as clinical manifestations, coupled with the use of chemotherapy drugs, further consume qi and injure blood, thereby aggravating the symptoms or leading to various complications. This makes the diagnosis of CIPN difficult and results in incomplete or poor-quality literature that may have been included in this study. Second, different types of external treatment, treatment frequency, duration, and other factors have caused heterogeneity among the various studies. Finally, the quality of the methodology and applied method reports included in this study were poor, thereby creating a risk of bias. If the allocation results were not hidden, there may have been a selection bias. If the blinding was not implemented, that is, the subjects, researchers, and testers were not blinded, selection, implementation, and measurement bias were likely (Liu, 2012).

## 6 Conclusion

The results of this study indicated that compared with other therapies, external treatment with CHM for CIPN has obvious advantages; it may significantly decrease the incidence of peripheral neurotoxicity and clinical symptoms and improve quality of life. These results may guide clinical practice. Due to the limitations of this study, further rigorously designed, large-scale, multicenter RCT trials are needed to fully evaluate the efficacy of CHM external treatment for CIPN to provide more reliable evidence.

## Data Availability

The raw data supporting the conclusion of this article will be made available by the authors, without undue reservation.
